# Sentinel node navigation surgery in esophageal cancer

**DOI:** 10.1002/ags3.12206

**Published:** 2018-09-05

**Authors:** Hiroya Takeuchi, Yuko Kitagawa

**Affiliations:** ^1^ Department of Surgery Hamamatsu University School of Medicine Hamamatsu Japan; ^2^ Department of Surgery Keio University School of Medicine Tokyo Japan

**Keywords:** esophageal cancer, micrometastasis, radioisotope, sentinel node

## Abstract

Over the last 20 years, the sentinel node (SN) concept has been widely applied to the surgical staging of both breast cancer and melanoma. However, the validity of this concept has been controversial for esophageal cancer, because SN mapping for esophageal cancer is not considered to be technically easy because of the complicated multidirectional lymphatic networks of the esophagus and mediastinum. Nevertheless, studies including meta‐analyses indicated that SN mapping may be feasible in early esophageal cancer. Transthoracic esophagectomy with three‐field lymphadenectomy was developed as a potential curative procedure for thoracic esophageal cancer. However, this highly invasive procedure might increase morbidity and reduce patients’ quality of life (QOL) after esophagectomy. Although further validation based on multicenter trials using the standard protocol of SN mapping for esophageal cancer is required, SN navigation surgery would enable us to carry out personalized and limited lymph node dissection which might reduce morbidity and maintain patients’ QOL.

## 1 INTRODUCTION

Esophageal cancer is the eighth most common cancer worldwide, affecting more than 450 000 people per year and for which the incidence is increasing.^1^ Esophageal cancer also has one of the highest malignant potentials of any tumor. Its primary therapy is selected according to histological type, tumor stage and location, and patient's performance status and comorbidities.^2^ Although the efficacy of chemotherapy or chemoradiotherapy (CRT) for esophageal cancer has been reported, esophagectomy remains the mainstay of potential curative treatment for esophageal cancer.^2^

Lymph node metastasis is known as one of the useful indicators for predicting the long‐term outcome of esophageal cancer. Lymph node metastasis is not a rare event in esophageal cancer, with its incidence, even in submucosal tumors, reaching 45%.^3^ Other specific characteristics of esophageal cancer are multidirectional lymphatic flow from the primary tumor site and widespread and random patterns of lymph node metastasis from the neck to the abdominal areas. In fact, it was reported that anatomical skip metastases to the second or third compartment of regional lymph nodes were found in more than half of esophageal cancer cases.^3^ Middle thoracic esophageal cancer metastasized widely to the lymph nodes located from the neck (26%) to the abdomen (39%).^3^ Considering these specific characteristics, total or subtotal esophagectomy with three‐field lymphadenectomy has been recognized as a standard procedure for esophageal squamous cell carcinoma (SCC) in Japan.^3^, ^4^ However, esophagectomy with three‐field lymphadenectomy is one of the most invasive procedures in gastrointestinal (GI) surgeries even by minimally invasive esophagectomy such as thoracoscopic and laparoscopic approaches. Postoperative complications and mortality are known to be remarkably increased by three‐field lymphadenectomy.^5^ Sentinel node (SN) mapping may play a significant role in reducing the need for the uniform application of the highly invasive surgery by providing personal information to allow modifications of the surgical procedure for esophageal cancer patients.

The SN is defined as the first lymph node(s) receiving lymphatic drainage from the primary tumor site.^6^ The SN is considered to be the first possible site of lymph node micrometastasis from the primary tumor. If the SN is recognizable and pathologically negative for cancer metastasis, unnecessary radical lymphadenectomy could be omitted. SN navigation surgery is a type of less invasive surgery with modified or minimized lymphadenectomy based on the diagnosis of SN metastasis.

Sentinel node mapping and biopsy were first applied to breast cancer and melanoma and subsequently attempted for other solid tumors including GI cancers.^6^, ^7^, ^8^, ^9^, ^10^ SN mapping and biopsy results in reducing postoperative complications as a result of unnecessary extended lymphadenectomy in patients SN negative for cancer metastasis.^6^, ^7^

Sentinel node mapping for esophageal cancer is technically difficult in comparison with that for gastric cancer.^10^ However, SN mapping and biopsy might become useful tools for the accurate intraoperative diagnosis of lymph node metastasis and modification of the surgical procedures in minimally invasive surgery in patients with early‐stage esophageal cancer.^11^

## 2 SENTINEL NODE MAPPING AND BIOPSY PROCEDURES IN ESOPHAGEAL CANCER

**Figure 1:**
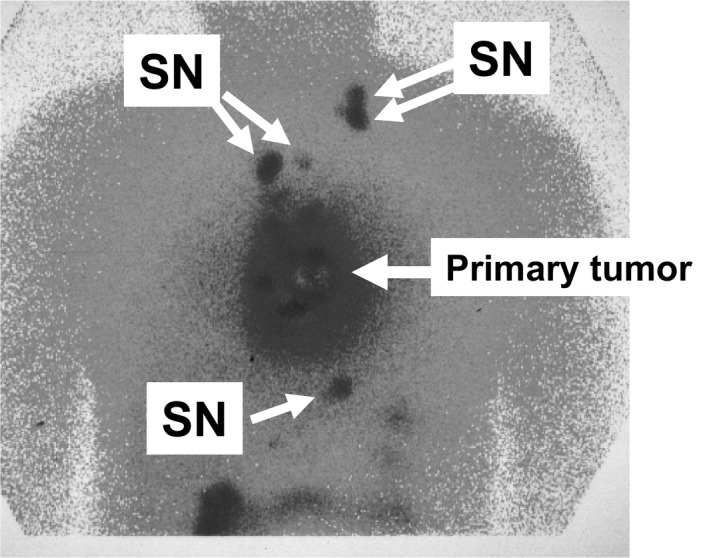
Preoperative lymphoscintigraphy in thoracic esophageal cancer. SN, sentinel node

A radio‐guided method is preferentially used to identify the SN in esophageal cancer.^10^, ^11^, ^12^ In our institution, technetium‐99*m* tin colloid solution as a radioisotope tracer is injected using an endoscopic puncture needle at four quadrants into the submucosal layer surrounding the primary tumor the day before surgery. Preoperative lymphoscintigraphy is usually carried out 3‐4 hours after injecting the tracer (Figure 1). In terms of the distribution of SN, they are spread widely from cervical to abdominal areas.

**Figure 2:**
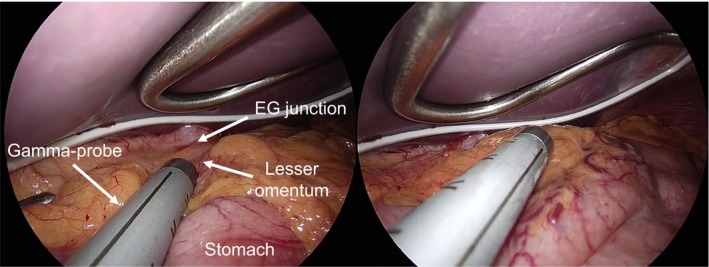
Intraoperative gamma probing during laparoscopic surgery. EG, esophagogastric

We usually use a handheld gamma probe to accurately identify SN, which can be introduced from the trocar ports for thoracoscopic or laparoscopic intraoperative SN mapping (Figure 2). SN located in the neck can be easily identified by percutaneous gamma probing. After intraoperative SN mapping and biopsy, all SN in the resected specimens are confirmed using the gamma probe on a back table near the operating table and sent for intraoperative pathological examination. Finally, we confirm the absence of residual SN in the mediastinum or abdominal cavity using the gamma probe. In order to verify the accuracy of the SN mapping, intraoperative SN mapping and biopsy are generally followed by total or subtotal esophagectomy with regional lymphadenectomy based on the Japanese guidelines.^12^

In contrast, a dual tracer method using radioactive tracer and blue dye (indocyanine green) is useful for SN detection for abdominal esophageal cancer or adenocarcinoma (AC) of the esophagogastric (EG) junction. The blue dye is injected into the submucosal layer around the primary tumor site endoscopically immediately after the start of surgery. Subsequently, the afferent lymphatics are visualized clearly even in laparoscopic observation, and blue‐stained nodes are identified as the SN approximately 15 minutes after the dye injection.

Dye‐only‐guided SN mapping is not recommended for thoracic esophageal cancer because lymph nodes in the mediastinum are frequently pigmented by anthracosis.^13^ Moreover, real‐time observation of the lymphatics using blue dye is sometimes difficult without operative mobilization of the esophagus, but the mobilization itself may disturb active lymphatic flow from the primary tumor site. However, the blue dye in addition to the radio‐guided method is useful in abdominal esophageal cancer or EG junction cancer, because it is relatively easy to identify blue‐stained lymphatic vessels and lymph nodes without the mobilization of the esophagus in the abdominal cavity compared with that in the mediastinum. Furthermore, pigmentation as a result of anthracosis is relatively rare in abdominal lymph nodes.

Endoscopic submucosal injection of tracer is useful for accurate SN mapping of esophageal cancer. The radioactive tracer, technetium‐99*m* tin colloid, has a relatively larger particle size (~200 nm in diameter) than blue dye.^14^ The radioactive tracer is known to migrate into the SN from the primary lesion within 2 hours after injection and accumulates in the SN without excessive diffusion. Radioactivity lasting at least 20 hours is sufficient for SN detection.^15^, ^16^ Preoperative lymphoscintigraphy is useful for detecting SN distant from the primary lesion before surgery (Figure 1). Furthermore, a handheld gamma probe is accurate and useful for intraoperative detection of SN in esophageal cancer.^12^ Gamma probing was shown to be feasible even in thoracoscopic or laparoscopic SN mapping.^12^

## 3 RESULTS OF SN MAPPING IN ESOPHAGEAL CANCER

To date, fewer studies have shown the feasibility and validity of the SN concept in esophageal cancer^10^, ^12^, ^13^, ^17^, ^18^, ^19^, ^20^, ^21^, ^22^, ^23^, ^24^, ^25^, ^26^, ^27^, ^28^, ^29^, ^30^ compared with studies on gastric cancer (Table 1). However, a number of single institution studies have indicated acceptable outcomes of SN mapping and biopsy for early‐stage esophageal cancer.^31^ In particular, a radio‐guided method appears to be superior regarding the SN detection rate and accuracy at predicting lymph node metastasis compared to the conventional dye‐guided method for esophageal cancer (Table 1).

**Table 1:** Representative results of sentinel node mapping in esophageal cancer

Author (Ref.)	Year	Tracer	Tumor depth	Number of patients	SN detection rate (%)	Sensitivity (%)	Accuracy (%)
Kitagawa^10^	2000	RI (^99m^Tc tin colloid)	cT1‐T3	27 SCC	25/27 (93)	14/16 (88)	23/25 (92)
Kato^17^	2003	RI (^99m^technetium rhenium sulfide)	pT1‐T4	25 SCC	23/25 (92)	13/15 (87)	21/23 (91)
Yasuda^18^	2003	RI (^99m^Tc tin colloid)	pT1‐T3	23 SCC + AC	23/23 (100)	9/12 (75)	20/23 (87)
Stein^19^	2004	RI (^99m^Tc colloid) + isosulfan blue	pT1‐T3	35 AC	32/35 (91)	2/4 (50)	30/32 (94)
Udagawa^20^	2005	RI (^99m^Tc tin colloid and ^99m^Tc phytate)	cT1	24 SCC	24/24 (100)	9/13 (69)	20/24 (83)
Lamb^21^	2005	RI (^99m^Tc nanocolloid)	ND	57 AC	57/57 (100)	35/37 (95)	55/57 (96)
Arima^22^	2006	RI (^99m^Tc tin colloid)	pT1‐T3	19 SCC	18/19 (95)	14/18 (78)	14/18 (78)
Takeuchi^12^	2009	RI (^99m^Tc tin colloid)	cT1‐T2	75 SCC + AC	71/75 (95)	29/33 (88)	67/71 (94)
Grotenhuis^13^	2009	Patent blue V	pT1‐T3	40 AC	39/40 (98)	27/33 (82)	33/39 (85)
Bhat^23^	2010	Methylene blue	pT1‐T3	32 SCC + AC	26/32 (81)	20/26 (77)	14/20 (70)
Thompson^24^	2011	RI (^99m^Tc antimony colloid)	pT1a‐T3	16 SCC + AC	14/16 (88)	3/3 (100)	16/16 (100)
Kim^25^	2011	RI (^99m^Tc neomannosyl HSA)	cT1‐T4	23 ESCC	21/23 (91)	8/8 (100)	21/21 (100)
Uenosono^26^	2011	RI (^99m^Tc tin colloid)	cT1‐T3	134 SCC + AC	cT1: 56/60 (93)	cT1: 11/12 (92)	cT1: 55/56 (98)
					cT2: 31/31 (100)	cT2: 12/18 (67)	cT2: 25/31 (81)
					cT3: 28/32 (88)	cT3: 13/24 (54)	cT3: 17/28 (61)
					CRT: 5/11 (46)	CRT: 0/3 (0)	CRT: 2/5 (40)
Yuasa^27^	2012	Indocyanine green fluorescence	cT1	20	19/20 (95%)	3/4 (75%)	18/19 (95)
Takeuchi^28^	2015	RI (^99m^Tc tin colloid)	pT1	70 SCC + AC	65/70 (93)	22/24 (92)	63/65 (97)
Boone^29^	2016	RI (^99m^Tc nanocolloid)	cT1‐T3	8 SCC + AC	8/8 (100)	0/5 (0)	3/8 (38)
Künzli^30^	2017	RI (^99m^Tc nanocolloid)	cT1	5 AC	5/5 (100)	0/1 (0)	4/5 (80)

AC, adenocarcinoma; CRT, chemoradiotherapy; HSA, human serum albumin; ND, not determined; RI, radioisotope; SCC, squamous cell carcinoma; SN, sentinel node; Tc, technetium.

Several types of radioisotope tracers have been used in SN studies for esophageal cancer (Table 1).^17^, ^21^, ^25^ Specifically, particle size of the radiocolloid is thought to be related to the time of tracer deposition in the lymph node; therefore, a larger particle such as tin colloid would be associated with a longer period of deposition. SN mapping with technetium‐99*m* colloidal rhenium sulfide was also reported to be useful to identify the lymphatic basin in 25 patients with esophageal cancer, and the results mostly matched those obtained with technetium‐99*m* tin colloid.^17^ However, further studies will be needed to ensure the optimal selection of radioisotope tracers.

Several contraindications against the recommendation of SN mapping in esophageal cancer have been reported.^11^ Patients with clinically apparent lymph node metastasis should be excluded from the indication for SN mapping because the purpose of SN mapping is to identify clinically undetectable lymph node involvement. Regarding tumor depth, clinically T1 esophageal cancers were reported to be suitable for SN mapping.^10^, ^11^, ^26^ In contrast, clinically T3/T4 tumors may cause higher false‐negative rates in SN mapping because original lymphatic drainage routes might be changed according to the tumor progression of esophageal cancer. In fact, SN mapping failure was more frequently reported in clinically T3/T4 tumors than in T1 tumors.^26^ Therefore, clinically T3 or T4 tumors should also be considered a contraindication for SN mapping in esophageal cancer.

We previously reported a feasibility study of radio‐guided SN mapping and biopsy for 75 patients with clinically T1N0 or T2N0 esophageal cancer.^12^ Our study demonstrated acceptable SN detection in 71 (95%) of the 75 patients and successful diagnostic accuracy (94%) of nodal metastasis based on SN status. Mean number of identified SN was 4.7 per case in our study, but SN mapping was feasible even during thoracoscopic esophagectomy.^12^ In our study, the distribution of SN showed a wide spread from cervical to abdominal areas.^12^ In upper thoracic esophageal cancer, SN were frequently identified in the lymph nodes along the bilateral recurrent laryngeal nerve chain (stations #106recR and #106recL in the Japanese guidelines) and lymph nodes in the cervical area. In middle thoracic esophageal cancer, a wide distribution of SN was shown from cervical to abdominal areas. In particular, SN were frequently identified in the stations #106recR and #106recL, bifurcational and main bronchus lymph nodes (stations #107 and #109), and middle thoracic paraesophageal nodes (station #108) in middle thoracic esophageal cancer. However, more than 10% of the patients with middle thoracic esophageal cancer showed SN in the area along the cardia and lesser curvature of the stomach (stations #1, #2, #3, and #7). SN were mainly located in the lower and middle mediastinum and in the abdominal area in lower thoracic esophageal cancer, but some patients showed SN in the upper mediastinum. In more than 85% of patients with thoracic esophageal cancer, at least one SN was found to be located in the second or third compartment of regional lymph nodes.^12^ Generally, the lymph node stations that were frequently identified as SN showed a tendency to have a high incidence of metastasis, as detected pathologically.

In another study, 70 patients who underwent radio‐guided SN mapping and who were diagnosed with pathological T1 primary thoracic esophageal cancer were assessed in our institution.^28^ Results showed that 85% of the patients had no lymph node metastasis or had lymph node metastasis in SN only. Moreover, disease‐specific survival of the patients with metastatic non‐SN was significantly worse than that of the patients with no lymph node metastasis or lymph node metastasis in SN only. This study demonstrated that SN mapping is useful not only as an accurate diagnostic tool for detecting lymph node metastasis but also for prognostic stratification in patients with cN0 early esophageal cancer.

In comparison with SCC, distribution of SN is relatively localized in the lower mediastinum and in the abdominal area in the AC, but a previous study reported a high false‐negative rate reaching 15% in SN mapping in the AC.^13^ However, the lower accuracy may result from several concerns such as dye‐only‐guided SN mapping, use of transhiatal approach, and many patients with T3 tumors (65%) in the study. In contrast, Lamb et al^21^ reported acceptable outcomes of SN mapping in 57 patients with AC. Another group also demonstrated that SN mapping is feasible and accurate in patients with AC of the EG junction.^32^ We consider that SN mapping and biopsy are also applicable to AC of the distal esophagus or EG junction.

Nagajara et al^31^ reported a systematic review and meta‐analysis that evaluated the diagnostic value of SN mapping in esophageal cancer. Results of their meta‐analysis, which included 23 relevant studies with 623 patients, indicated that the SN detection rate and the accuracy of prediction of lymph node metastasis based on SN status were 93% and 88%, respectively, and were not markedly different between SCC and AC. Although they concluded that SN mapping is technically feasible in esophageal cancer, they also reported that the detection rate and accuracy were higher in radioisotope tracer in comparison with blue dyes.^31^

In contrast, in their meta‐analysis including 18 studies, Kakhki et al^33^ reported that the sensitivity to predict nodal metastasis was 84% in total and markedly higher for AC compared to SCC (91% vs 81%). Also, they indicated that the sensitivity tended to be lower according to tumor depth (T1: 88%, T2: 76%, T3: 50%) in the SCC patients. They concluded that cN0 patients with superficial primary tumors (T1 or T2) would be suitable for SN mapping for esophageal SCC.^33^

In general, distribution of nodal metastasis even after neoadjuvant therapy such as neoadjuvant chemotherapy or CRT could be useful information in the pathological staging of disease and in the planning of surgical procedures. Thompson et al^24^ found no remarkable differences in identifying SN between patients with and without neoadjuvant therapy. However, Uenosono et al^26^ reported that SN mapping is unacceptable for patients who have received neoadjuvant CRT because of the high false‐negative rate.

Recently, the utility of single‐photon emission computed tomography/computed tomography (SPECT/CT) for SN mapping has been reported in various solid tumors including esophageal cancer.^34^, ^35^ SPECT/CT for SN mapping could provide precise localization with 3‐D imaging compared to conventional lymphoscintigraphy. In contrast, Yuasa et al^27^ attempted intraoperative indocyanine green fluorescence imaging for SN biopsy in early‐stage esophageal cancer combined with preoperative CT lymphography. Although further studies will be required to verify the clinical utility of these new methods, in the future, technical innovations may improve the accuracy of SN mapping in esophageal cancer.

## 4 CLINICAL SIGNIFICANCE OF MICROMETASTASIS IN SN OF ESOPHAGEAL CANCER

Even now, positron emission tomography (PET)/CT scans still have limited sensitivity in detecting micrometastasis in esophageal cancer compared with conventional pathological examinations.^36^, ^37^, ^38^ Although SN mapping in esophageal cancer is clearly more invasive than other imaging, SN mapping and biopsy are believed to detect micrometastasis more accurately than other imaging procedures in patients with cN0 early‐stage esophageal cancer.^28^, ^39^

Several studies have demonstrated underestimation of the frequency of micrometastasis in regional lymph nodes by conventional pathological diagnosis using hematoxylin and eosin staining.^40^ In addition, immunohistochemical nodal micrometastasis is reported to be a significant prognostic indicator in patients with cN0 esophageal cancer.^41^, ^42^, ^43^ However, immunohistochemical examinations for all resected lymph nodes by esophagectomy are not practically feasible in clinical practice. SN mapping and biopsy can easily focus on the diagnosis of micrometastasis by assessing the small number of lymph nodes using immunohistochemistry. Immunohistochemistry and reverse transcription polymerase chain reaction (RT‐PCR) were reported to improve the sensitivity of SN mapping for predicting nodal metastasis in patients with esophageal cancer.^26^, ^44^ However, the clinical utility of RT‐PCR in SN of patients with early‐stage esophageal cancer remains controversial regarding its clinical impact, as well as its time‐ and labor‐consuming characteristics, and cost‐effectiveness.^45^ Further studies are required to evaluate the clinical significance of micrometastasis detected by RT‐PCR in SN in patients with early‐stage esophageal cancer.

## 5 FUTURE PERSPECTIVES OF SN MAPPING IN ESOPHAGEAL CANCER

Radical esophagectomy with extended three‐field lymphadenectomy has been established as a potential curative surgical procedure for thoracic esophageal SCC in Japan.^1^, ^2^ Esophagectomy with three‐field lymphadenectomy may be reasonable on account of the wide distribution of SN and unpredictable metastatic nodes.^12^, ^46^ Regarding this point, the clinical application of SN mapping and biopsy in esophageal cancer may be unexpectedly limited. However, uniform application of esophagectomy with three‐field lymphadenectomy might increase the postoperative complications and remarkably reduce quality of life (QOL) after surgery. SN mapping and biopsy may provide significant information to carry out personalized selective lymph node dissection that could reduce postoperative complications without a negative impact on the prognosis.^47^ For example, if SN were identified in the mediastinum or abdominal area only and all SN were pathologically negative for cancer metastasis in patients with cT1N0 middle or lower thoracic esophageal cancer, cervical lymphadenectomy would be omitted.^12^ However, we also consider that SN mapping and biopsy will even be applicable to two‐field lymphadenectomy and provide useful information regarding the extent of lymphadenectomy.^12^ For instance, if SN is identified along the recurrent laryngeal nerves in upper mediastinum and is positive for cancer metastasis, extended lymphadenectomy for the upper mediastinum might be considered even in Ivor Lewis esophagectomy.

SN mapping and biopsy will also be applicable to AC of the distal esophagus or EG junction to adjust and modify the surgical procedures.^12^ For instance, if all SN were identified in the abdominal area only and were negative for metastasis by intraoperative pathological diagnosis in a patient with cT1N0 AC of distal esophagus, the patient could be treated with limited resection of the distal esophagus by a laparoscopic transhiatal approach without extensive mediastinal lymphadenectomy.^28^, ^48^ In contrast, if the SN(s) were positive for metastasis, the patient should be treated with thoracoscopic mediastinal lymphadenectomy.^49^ Although we need to evaluate the survival benefit of modified lymph node dissection based on the SN concept, the new surgical procedure might reduce the morbidity and mortality without a negative impact on the QOL for early esophageal cancer patients with pathologically negative SN.

Definitive CRT was reported to be a good option for patients with cT1N0M0 esophageal SCC who do not want surgical treatment.^50^ However, how to design the optimal irradiation field is still controversial, because larger fields might be better regarding control of subclinical lymph node metastasis, but may result in more adverse effects during long‐term follow up.^51^ We have carried out concurrent CRT for 16 patients with cT1N0M0 esophageal SCC with minimized irradiation fields that contain SN identified by lymphoscintigraphy to achieve local control of subclinical metastasis in SN.^52^ Of the 16 patients, there were nine cases of definitive CRT and seven of adjuvant CRT after endoscopic resections. The results showed that all patients with definitive CRT achieved complete remission. At the median follow up of 77 months, there was no lymph node recurrence. Prophylactic irradiation of the hot spots in lymphoscintigraphy may be effective for controlling subclinical micrometastasis in SN, although further evaluation concerning the validity of irradiation field settings based on lymphoscintigraphy is needed in patients with superficial esophageal SCC.

**Figure 3:**
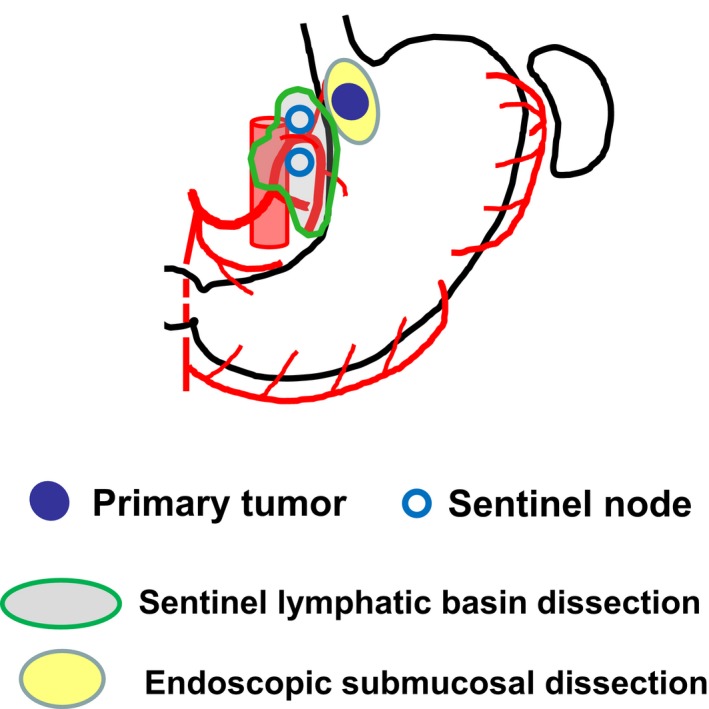
Endoscopic submucosal dissection combined with sentinel node mapping and biopsy in cT1bN0M0 esophagogastric junction cancer

In addition, further investigations should clarify the feasibility and efficacy of novel esophagus‐preserving treatments, such as endoscopic submucosal dissection in patients with cT1b esophageal SCC or in EG junction cancer patients having pathologically negative SN confirmed by thoracoscopic or laparoscopic SN mapping and biopsy (Figure 3). However, the details of SN mapping techniques such as the choice of tracers and the timing of tracer injection need to be standardized and evaluated carefully for the clinical application of SN mapping and biopsy in early‐stage esophageal cancer. Until now, no multicenter prospective trials have been conducted to validate the SN concept in esophageal cancer because a standard protocol for SN mapping procedures has not yet been established. Accumulation of evidence based on multicenter prospective trials would be expected to verify the feasibility and reliability of SN mapping in early esophageal cancer.

**Figure 4:**
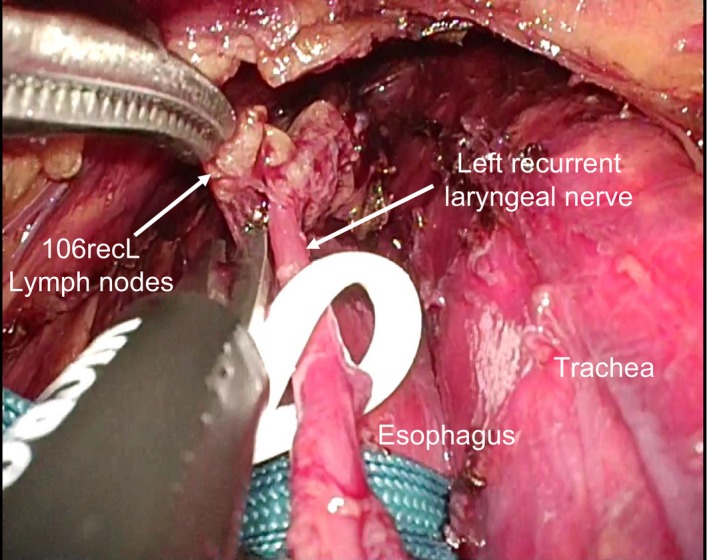
Mediastinoscopic sampling of lymph node along the left recurrent laryngeal nerve (#106recL) from the left cervical incision

Moreover, further improvement in surgical approaches should be achieved for less invasive and more accurate SN mapping. Recently, Fujiwara et al^53^ developed a lymphadenectomy method in the left upper mediastinum using a left cervical approach with a single‐port mediastinoscopic technique. The new surgical techniques enable us to access #106recL easily without mobilization of the esophagus by a right thoracoscopic approach (Figure 4). The combination of the mediastinoscopic SN mapping and biopsy techniques might revolutionize SN navigation surgery for early‐stage esophageal cancer.

## 6 CONCLUSION

The incidence of lymph node metastasis is relatively high even in cN0 early‐stage esophageal cancer. Therefore, SN mapping must be accurate for carrying out SN navigation surgery in esophageal cancer. Previous studies suggested that the SN concept appears to be valid and radio‐guided SN mapping may be feasible in cT1N0 esophageal cancer. Further accumulation of evidence based on multicenter trials using a standard protocol of SN mapping is needed, but SN navigation surgery will become an ideal less invasive personalized treatment for early‐stage esophageal cancer.

## DISCLOSURE

Conflicts of Interest: Authors declare no conflicts of interest for this article.

Author Contributions: HT wrote the manuscript and made tables and figures. YK supervised the whole editing.
